# Ruptured gastric artery aneurysms: two cases and emergency imaging findings

**DOI:** 10.1259/bjrcr.20170075

**Published:** 2017-11-10

**Authors:** Simon McQueen, John Vedelago, John Velkovic, Mark Page, Elizabeth Dick

**Affiliations:** ^1^Emergency Medicine, Coffs Harbour Base Hospital, Coffs Harbour, Australia; ^2^Everlight Radiology, London, UK; ^3^Department of Radiology, Sunshine Coast University Hospital, Queensland, QLD, Australia; ^4^Radiology, Everlight Radiology, Tel Aviv, Israel; ^5^Department of Radiology, St Mary’s Hospital, London, UK

## Abstract

A ruptured gastric artery aneurysm is a rare but important possible cause of massive intra-abdominal or gastrointestinal haemorrhage, and carries a high risk of mortality. Although aneurysms of the gastric arteries are uncommon, emergency radiologists and clinicians should be familiar with the clinical presentation, imaging findings and pathophysiology. We present two cases of massive intra-abdominal haemorrhage and haemodynamic shock secondary to acute rupture of previously occult gastric artery aneurysm and review the relevant anatomy, imaging findings and pathophysiology of gastric and other visceral artery aneurysms. By virtue of its location in the lesser omentum, a ruptured gastric artery aneurysm may result in a typical pattern and distribution of adjacent haematoma in the upper abdomen. Our description of imaging findings highlights a characteristic epicentre of intraperitoneal haemorrhage, and its typical mass effect displacement of surrounding viscera, to aid the emergent diagnosis of gastric artery aneurysm rupture.

## Case series

### Case One

A 51-year-old female presented to the emergency department with sudden onset of epigastric pain and vomiting. There was no known history of visceral or other arterial aneurysm, pancreatitis or connective tissue disease and the patient was otherwise fit and well. On examination, she was hypotensive (85/60 mmHg), with rebound and four quadrant abdominal tenderness.

**Figure 1.**
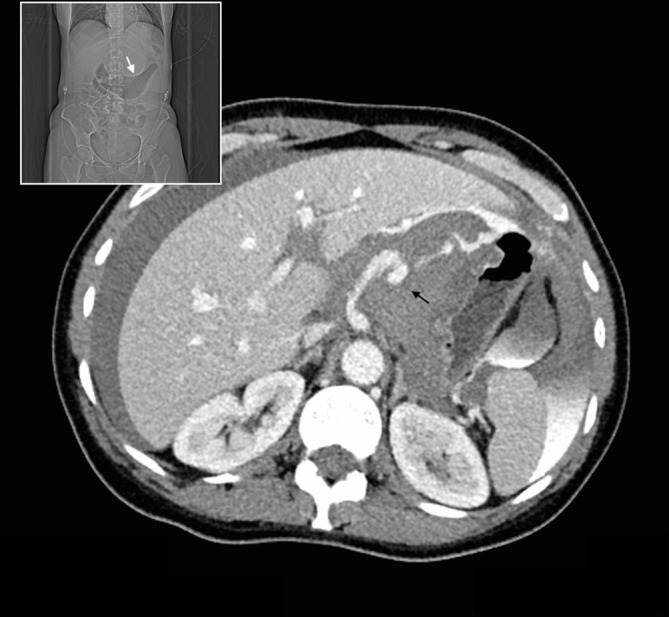
Ruptured bilobed 18 mm left gastric artery aneurysm with extravasation of contrast media (black arrow); the bulbous saccular nature of contrast enhancement is suggestive of aneurysm here (compared to the more linear appearance of active haemorrhage extending from this location). Associated anteroposterior scout image shows inferiorly displaced lesser curvature of the stomach (white arrow).

**Figure 2.**
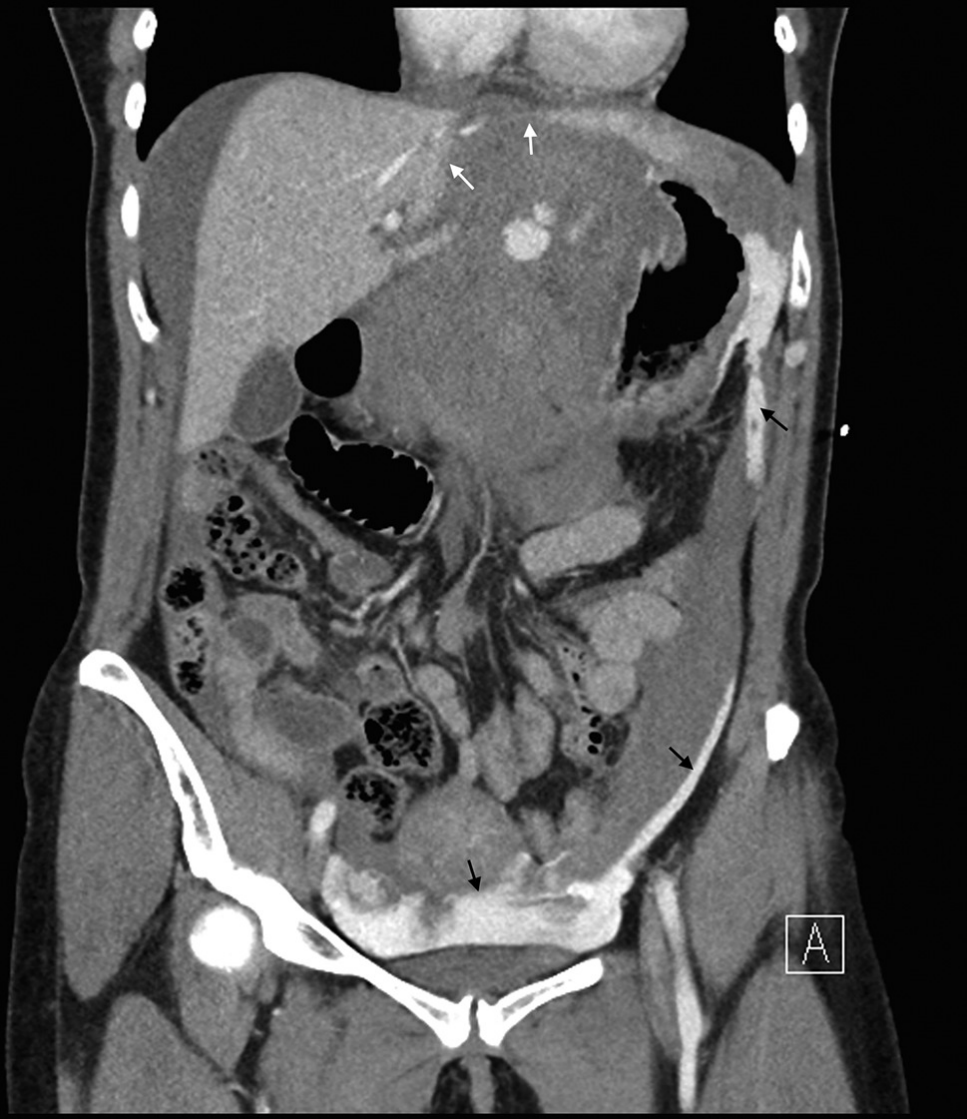
Widespread contrast media leak within the peritoneal cavity. Black arrows indicate blood in the pelvis and left paracolic gutter. White arrows indicate superior displacement of the left lobe of the liver.

**Figure 3.**
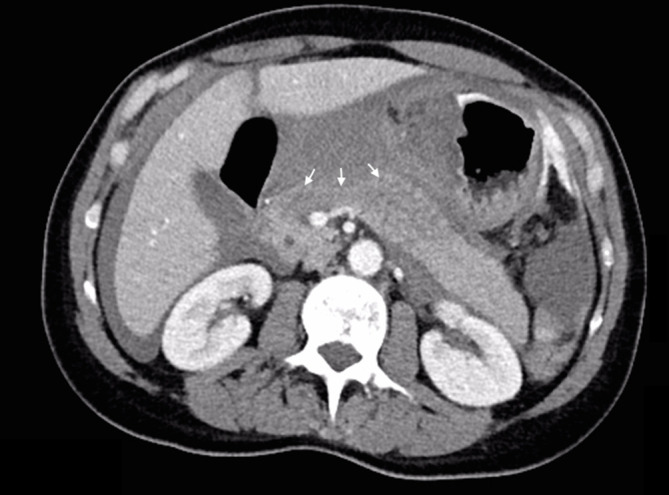
Sentinel clot displacing the pancreas head and body posteriorly (arrows).

**Figure 4.**
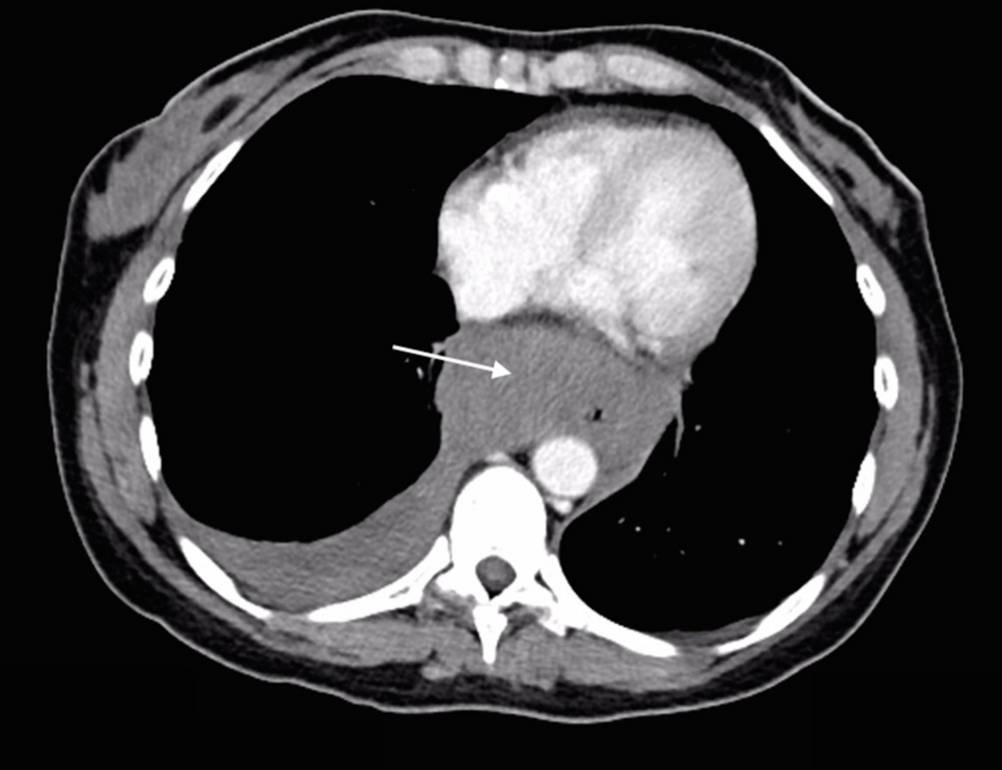
Haematoma (white arrow) has expanded the oesophageal hiatus, extending in to the chest cavity, compressing the oesophageal lumen and pooling posterior to the right lung. Pleural effusion is present.

Urgent portal venous phase abdominal CT was performed. The anteroposterior scout image showed a large epigastric density displacing the lesser curvature of the stomach inferiorly (Figure 1 white arrow). Post administration of contrast, images showed extremely rapid extravasation of contrast from a ruptured bilobed 18 mm left gastric artery aneurysm (Figure 1 black arrow). From the time of contrast injection to the time of imaging (approximately 70 s) a very large volume of contrast had extravasated into the peritoneal cavity, and was evident in the pelvis, left paracolic gutter (Figure 2 black arrows) and lesser sac, confirming very rapid bleeding. An expanded haematoma was present and centred in the region of the lesser omentum and lesser sac. This haematoma displaced the gastric lesser curvature inferiorly, the pancreatic head and body posteriorly (Figure 3) and the underside of the left lobe of the liver superiorly. The haematoma was seen extending through the oesophageal hiatus, which was also expanded (Figure 4). There were signs of hypoperfusion complex with flattened inferior vena cava and adrenal hyperattenuation. The patient underwent urgent surgery during which the aneurysm was clipped and resected, and she made an uneventful recovery.

### Case Two

A 57-year-old female presented with abdominal pain radiating to the back, with presyncopal symptoms. There was no known relevant history. Urgent triple phase CT demonstrated a right gastric artery aneurysm, located near the region of its anatomical anastomosis with the left gastric artery. No definite active haemorrhage was seen at the time of the study. A very large volume haemoperitoneum was present. A haematoma with epicentre at the lesser omentum/lesser sac was present. The haematoma displaced the lesser curvature of the stomach inferolaterally and filled the lesser sac. There was mild compressive mass effect on the anterior aspect of the pancreas. Emergent surgery was performed, and the aneurysm oversewn. The patient made complete recovery.

## Discussion

Although gastric artery aneurysm rupture as a presentation is uncommon as gastric artery aneurysms account for just 4% of all visceral artery aneurysms,^1^ rupture is associated with a high mortality rate.^2^

The left gastric artery originates from the coeliac axis, the first major visceral branch of the abdominal aorta. Noting that there is a considerable degree of anatomic variation of the coeliac axis and its branches, in conventional anatomic description the coeliac axis divides at the upper border of the pancreas into the left gastric artery, and then bifurcates to form the common hepatic and splenic arteries. The left gastric artery originates directly from the abdominal aorta in 2–3% of cases.^3^ The left gastric artery supplies the superior portion of the lesser curvature of the stomach, sending branches to the intra-abdominal portion of the distal oesophagus.

The right gastric artery arises from the hepatic artery proper in 53% of the population, at the division of the common hepatic artery (20%), as a branch of the left hepatic artery (15%), from the gastroduodenal (8%) or as a branch of the common hepatic artery (4%).^4^ The right gastric artery supplies the inferior portion of the lesser curvature of the stomach. Along the lesser curvature of the stomach, the right and left gastric arteries may run as single or double vessels, and share an end to end anastomosis.^3^

The lesser omentum, within which the gastric arteries are invested, develops as the dorsal portion of the embryologic ventral mesogastrium. It is a continuation of the peritoneal layers overlying the anterosuperior surface of the stomach and first part of the duodenum and the posteroinferior aspects of these structures, and is divided into gastrohepatic and hepatoduodenal parts accordingly. The right and left gastric arteries run between these peritoneal layers of the lesser omentum. As the layers ascend towards the porta hepatis they attach to the fossa for the ductus venosus, and at the diaphragm envelop the distal oesophagus after separating.^5^

The course of the gastric arteries through the gastrohepatic part of the lesser omentum explains both the location of the sentinel clot on these studies and the direction of displacement and mass effect on adjacent visceral structures. The presence of an expanded blood haematoma epicentred at the expected region of the lesser omentum and lesser sac and accompanying imaging features such as displacement of the lesser curvature of the stomach inferiorly or inferolaterally, displacement of the left lobe of the liver superiorly and compressive posterior mass effect on the anterior aspect of the pancreas were features common to both cases, and consistent with a focus of haemorrhage centred in the lesser omentum. In Case 1, there was also a large volume of haematoma extending through and expanding the oesophageal hiatus.

Pathologies implicated in the aetiology of gastric artery aneurysms include atherosclerosis, medial wall degeneration/dysplasia, infection or inflammation/vasculitides as well as an association with a variety of conditions including Marfan’s, Ehlers–Danlos and Osler–Weber–Rendu syndromes, fibromuscular dysplasia, Kawasaki disease and hyperflow conditions such as portal hypertension and pregnancy.^6,7^ Pseudoaneurysm can result from a partial tear through the vessel wall, which may be due to iatrogenic injury or trauma. Periarterial inflammation secondary to prior pancreatitis or hepatitis^2^ has been theorized as a possible contributory factor. The patients presented in this series had no known history of any of the aforementioned conditions.

In the setting of acute rupture with haemodynamic instability, open surgical management of visceral artery aneurysms is regarded by many authors as the treatment of choice.^8^ Surgical treatment options include open or laparoscopic excision of the aneurysm. Vascular perfusion may or may not be re-established, or the affected organ removed (*i.e.* splenectomy).^9^

Current treatment options for non-ruptured gastric artery aneurysms include open and endovascular techniques; however, there are no uniform consensus guidelines for management. Endovascular repair offers the potential advantage of a less invasive procedure than open surgical techniques. Potential complications of endovascular repair include distal thromboembolic event, non-target vessel embolization, coil migration, end-organ infarction and intraprocedural aneurysm rupture. For non-ruptured visceral artery aneurysms, it has been suggested that all pseudoaneurysms warrant consideration for treatment^6^ as do all symptomatic true aneurysms.^9^ Treatment has been suggested as potentially indicated for non-symptomatic aneurysms that have a diameter greater than 2 cm or are increasing at a rate greater than 0.5 cm/year.^10^

In the follow-up of a patient with a gastric artery aneurysm, complete abdominal imaging incorporating arterial phase cross-sectional imaging may be of value, as a single visceral artery aneurysm may be associated with other visceral artery aneurysms.^7^ The two patients featured in this case report had no evidence of further visceral aneurysm on follow-up imaging.

## Learning points

1. Although uncommon, in a clinical presentation of acute abdominal pain and hypotension, the possibility of ruptured gastric artery aneurysm should be considered in the differential diagnosis. Mortality if ruptured is high.
2. Ruptured gastric artery aneurysms may or may not show active haemorrhage at CT in the presence of a large volume haemoperitoneum.
3. The presence of an expanded sentinel clot that appears epicentred in the lesser omentum and lesser sac, thereby compressing the anterior aspect of the pancreas, displacing the left lobe of the liver superiorly and the lesser curvature of the stomach inferolaterally may provide useful clues to the diagnosis.

## Consent

Written informed consent for the case to be published (including images, case history and data) was obtained from the patient(s) for publication of this case report, including accompanying images.
